# Anti-inflammatory effects induced by ultralow concentrations of bupivacaine in combination with ultralow concentrations of sildenafil (Viagra) and vitamin D3 on inflammatory reactive brain astrocytes

**DOI:** 10.1371/journal.pone.0223648

**Published:** 2019-10-09

**Authors:** Elisabeth Hansson, Eva Skiöldebrand

**Affiliations:** 1 Department of Clinical Neuroscience, Institute of Neuroscience and Physiology, The Sahlgrenska Academy, University of Gothenburg, Gothenburg, Sweden; 2 Department of Pathology, Institute of Biomedical Sciences and Veterinary Public Health, Swedish University of Agricultural Sciences, Uppsala, Sweden; 3 Department of Clinical Chemistry and Transfusion Medicine, Institute of Biomedicine, Sahlgrenska University Hospital, Gothenburg University, Gothenburg, Sweden; Hungarian Academy of Sciences, HUNGARY

## Abstract

Network coupled cells, such as astrocytes, regulate their cellular homeostasis via Ca^2+^ signals spread between the cells through gap junctions. Intracellular Ca^2+^ release is controlled by different signaling pathways that can be stimulated by ATP, glutamate and serotonin (5-HT). Based on our findings, all these pathways are influenced by inflammatory agents and must be restored to fully recover the Ca^2+^ signaling network. An ultralow concentration of the local anesthetic agent bupivacaine reduced 5-HT-evoked intracellular Ca^2+^ release, and an ultralow concentration of the phosphodiesterase-5 inhibitor sildenafil in combination with vitamin D3 reduced ATP-evoked intracellular Ca^2+^ release. Combinations of these three substances downregulated 5-HT-, glutamate- and ATP-evoked intracellular Ca^2+^ release to a more normal Ca^2+^ signaling state. Furthermore, inflammatory Toll-like receptor 4 expression decreased with a combination of these three substances. Substance P receptor neurokinin (NK)-1 expression was reduced by ultralow concentrations of bupivacaine. Here, bupivacaine and sildenafil (at extremely low concentrations) combined with vitamin D3 have potential anti-inflammatory properties. According to the present study, drug combinations at the right concentrations, especially extremely low concentrations of bupivacaine and sildenafil, affect different cellular biochemical mechanisms and represent a potential solution for downregulating inflammatory parameters, thereby restoring cells or networks to normal physiological homeostasis.

## Introduction

Gap junction-coupled cell networks can be targets when the body or different organs are exposed to inflammatory stimuli, such as bacteria or viruses [1,2]. Astrocytes in the central nervous system (CNS) have been studied for several decades regarding their physiological and network coupling properties. These cells control extracellular and intracellular homeostasis at all levels of the CNS and may also contribute to the homeostasis of the other nervous systems in the body [3,4]. The strategic organization of astrocytes with their long processes and end feet, which make contact with other cellular networks, barriers, ventricles, etc., play an important role in chronic neuroinflammation [5,6]. During inflammation, the expression and affinities of several receptors are changed [7–9], the cytoskeleton is disrupted, and Ca^2+^ signaling is elevated, resulting in increased adenosine triphosphate (ATP) production, thereby changing the balance of Ca^2+^-regulating processes [10,11]. This change in Ca^2+^ signaling causes reduced communication between cells via gap junctions [12]. Furthermore, Na^+^ transporters are downregulated at the cellular level [13], and increased release of proinflammatory cytokines is observed [7].

Inducers of inflammation trigger the production of inflammatory mediators, which alter the functionality of connective tissues and organs and lead to harmful induction of different barrier systems, such as the blood-brain barrier (BBB), blood-nerve barrier, and blood-lymph barrier [14,15]. Bupivacaine, a local anesthetic, has been proposed to attenuate inflammatory responses at concentrations much lower than those that block Na^+^ channels [16,17]. Bupivacaine decreases the expression of nuclear factor (NF)-қB and proinflammatory cytokines such as tumor necrosis factor-α (TNF-α), interleukin-1β (IL-1β) and interleukin-6 (IL-6) [18]. Bupivacaine treatment in rat models of inflammatory pain decreased the activation of microglia and astrocytes [18].

Toll-like receptors (TLRs) trigger innate immune responses and stimulate glial cells to release proinflammatory mediators and cytokines [19]. TLR4 is present on astrocytes and shows increased expression after lipopolysaccharide (LPS) induction [7]. The active form of vitamin D3, 1α,25-dihydroxyvitamin D3 (1,25(OH)_2_D_3_), can access the CNS via passive and/or active transport across the BBB. Vitamin D3 stimulates its receptor, vitamin D receptor (VDR), on all neuronal cells, including astrocytes [20]. Furthermore, vitamin D3 has neuroprotective roles through its influence on the expression of nitric oxide synthase (iNOS), which in turn affects nitric oxide (NO) [20], decreases TLR4 expression on astrocytes [21,22] and reduces BBB damage [23].

Ca^2+^ influx over the plasma membranes can result in increased concentrations of cyclic guanosine monophosphate (GMP), which activates protein kinase G (PKG) [24]. However, cyclic GMP is rapidly hydrolyzed by phosphodiesterases (PDEs), among which PDE-5 seems important [25]. PDE inhibitors exert a direct anti-inflammatory effect by increasing cyclic GMP levels. [25–27]. The potent and selective PDE-5 inhibitor sildenafil increases GMP concentrations, which can ameliorate the effect of inflammation [27]. We previously demonstrated that sildenafil attenuates ATP-evoked intracellular Ca^2+^ release in LPS-induced inflammatory reactive astrocytes [28].

We also studied another combination of substances at extremely low concentrations where sildenafil was included. In this combination, in addition to sildenafil, extremely low concentrations of the μ-opioid antagonist naloxone, a μ-opioid agonist and the anti-epileptic drug levetiracetam were evaluated [29]. We believe that this combination [29], which affects glutamate- and ATP-evoked Ca^2+^ signaling, may have positive effects on neuroinflammation, while the combination that is the focus of the present study has wider anti-inflammatory properties.

The purpose of the present study was to attenuate inflammation-induced LPS effects on astrocytes to return the cells to a more physiological state with a drug combination that primarily affects 5-HT- and ATP-evoked Ca^2+^ signaling. At ultralow concentrations, bupivacaine and sildenafil attenuate 5-HT-evoked intracellular Ca^2+^ release [17] and ATP-evoked intracellular Ca^2+^ release, respectively [28]. We hypothesized that a combination of bupivacaine, sildenafil (both at ultralow concentrations), and vitamin D3 under high-glucose conditions exerts better anti-inflammatory properties than the individual substances. A high-glucose concentration might also have some anti-inflammatory properties [29,30].

## Materials and methods

### Cell model system

Rat primary cortical astrocytes from newborn Sprague-Dawley rats were purchased from 3H Biomedical Science (Uppsala, Sweden) and prepared according to the manufacturer’s instructions with some modifications [7,8,29]. The cultures were delivered in 5.5 mM glucose and cultivated in 5.5 mM glucose (physiological concentration) for the entire period.

### LPS treatment and pharmaceutical restoration

For anti-inflammatory restoration, the cell cultures were incubated with LPS (10 ng/ml) for 24 h. For restoration, inflammatory reactive cells were incubated with pharmaceutical substances together with LPS and 25 mM glucose for an additional 24 h. The substances used were bupivacaine (Marcain) (Astra Zeneca, Södertälje, Sweden) (10^−12^ M) [17], sildenafil citrate salt (Sigma Aldrich, St. Louis, MO, USA) (1 μM) [28] and 1α,25-dihydroxyvitamin D3 (calcitriol) (Sigma Aldrich) (100 nM) [31].

### Calcium imaging

With a high-throughput screening system for intracellular Ca^2+^ signaling, a Flexstation 3 Microplate Reader (Molecular Devices, San José, CA, USA), cells were incubated with the Ca^2+^-sensitive probe FLIPR Calcium 6 (Molecular Devices) and exposed to different neurotransmitters: 5-HT (10^−5^ M), glutamate (10^−3^ M), or ATP (10^−4^ M), all from Sigma Aldrich. The total areas under the curve (AUCs), which reflect the amount of Ca^2+^ released [32], were analyzed to measure the Ca^2+^ responses. The amplitude (peak) is expressed as the maximum increase.

### Protein determination

The protein determination assay was performed in accordance with the manufacturer’s instructions using a detergent compatible (DC) Protein Assay (Bio-Rad, Hercules, CA, USA) based on the method used by Lowry et al. [33] with minor modifications. The standard (0–4 mg/ml bovine serum albumin, BSA) and samples were mixed with the reagents, incubated for 15 min at room temperature, read at 750 nm with a Versa-max microplate reader, and analyzed using SoftMax Pro 4.8 from Molecular Devices (Sunnyvale, CA, USA).

### SDS-PAGE and western blotting

Cells were rinsed twice in phosphate-buffered saline (PBS) and immediately lysed for 20 min on ice in cold radio-immunoprecipitation assay (RIPA) lysis buffer containing 150 mM NaCl, 1% IGEPAL® CA-630, 0.5% sodium deoxycholate, 0.1% sodium dodecyl sulfate (SDS), and 50 mM Tris (pH 8.0) supplemented with a protease inhibitor cocktail containing 104 mM 4-(2-aminoethyl)benzenesulfonyl fluoride hydrochloride (AEBSF), 80 μM aprotinin, 4 mM bestatin, 1.4 mM E-64, 2 mM leupeptin, and 1.5 mM pepstatin A. The procedure was performed according to Persson et al. [34]. Separate aliquots were collected to determine the protein concentration. All of the samples were analyzed for the total protein content, and 20 μg of the total protein from each sample was loaded into each lane of the gel. β-Actin was used as a control for equal loading.

Sodium dodecyl sulfate-polyacrylamide gel electrophoresis (SDS-PAGE) was performed using a Novex precast gel system (Invitrogen, Carlsbad, CA, USA) according to the manufacturer’s recommendations using 4–12% Bis-Tris gels (Invitrogen) at 200 V for 50 min. The separated proteins were transferred at 30 V for 60 min to a nitrocellulose membrane (Invitrogen) using NuPAGE transfer buffer (Invitrogen) supplemented with methanol and NuPAGE antioxidant. The membranes were rinsed twice with distilled water, and the proteins were visualized with Ponceau S solution (Sigma Aldrich). The proteins were blocked with 0.5% fat-free skim milk (Semper AB, Götene, Sweden) in Tris-buffered saline (TBST; 50 mM Tris-HCl, 150 mM NaCl, and 0.05% Tween) for 60 min at room temperature. The membranes were probed with anti-TLR4 (rabbit polyclonal, 1:500) (Santa Cruz Biotech Inc, Dallas, TX, USA) or anti-neurokinin (NK)-1 (rabbit polyclonal, 1:1000) (LifeSpan BioSciences Inc, Seattle, CA, USA) antibodies or a mouse monoclonal primary antibody against Na^+^/K^+^-ATPase (α-subunit) (Sigma Aldrich) diluted 1:250 and washed 4 x for 2 min with TBST, followed by incubation with secondary horseradish peroxidase (HRP)-conjugated antibodies, donkey anti-mouse or anti-rabbit F(ab’)_2_ fragments (Jackson ImmunoResearch, Cambridge, UK) diluted 1:10000 and washed several times in TBST. All primary and secondary antibodies were diluted in 0.5% fat-free skim milk in TBST. The antibody-bound protein was detected with an enhanced chemiluminescence kit (PerkinElmer Inc., Waltham, MA, USA) and visualized using Fuji Film LAS-3000 (Tokyo, Japan).

### Immunocytochemistry

The cells were fixed with 4% paraformaldehyde (Bie & Berntsen, Herlev, Denmark) for 10 minutes and washed twice with phosphate buffer saline (PBS) (Invitrogen, Carlsbad, USA) containing 1% BSA (PBS-BSA). The cells were permeabilized with PBS-BSA containing 0.05% saponine (PBS-BSA-Sap) for 20 min. Thereafter the cells were incubated for 1 h with a cocktail of rabbit polyclonal antibody against glial fibrillary acidic protein (GFAP) (Dako, Glostrup, Denmark) and a mouse monoclonal antibody against OX42 (Serotec Oxford, UK). Both antibodies were diluted 1:100 in PBS-BSA-Sap. The cells were washed with PBS-BSA-Sap for 3 x 5 min and then incubated with a mixture of FITC conjugated F(ab´)_2_ fragment donkey anti-mouse IgG and a Dylight 594 conjugated F(ab´)_2_ fragment donkey anti-rabbit IgG secondary antibodies (JacksonImmuno Research Europe Ltd, Suffolk, UK), both diluted 1:150. The cells were washed with PBS-BSA-Sap for 3 x 5 min and finally rinsed with PBS. The cover slips were mounted on microscope slides with a fluorescent mounting medium (Dako) and viewed in a Nikon Eclipse 80*i* microscope. Pictures were taken with a Hamamatsu C5810 colour intensified 3CCD camera.

### Actin visualisation

The astrocyte cytoskeleton was stained with an Alexa^TM^488-conjugated phalloidin probe (Invitrogen). The cultures were fixed with 4% paraformaldehyde and made permeable with PBS (Invitrogen) containing 1% bovine serum albumin (BSA) and 0.05% saponin followed by an Alexa^TM^568-conjugated phalloidin probe (Invitrogen) diluted 1:40 in PBS supplemented with 1% BSA. The coverslips were rinsed three times in PBS and mounted on microscope slides using Dako’s fluorescent mounting medium (Dako) before being viewed with fluorescence dry-objective lens attached to an inverted Nikon Optiphot-2 microscope.

### Cytokine release

TNF-α (BD Biosciences, San Diego, USA) was used according to the manufacturer’s instructions to measure the amount of cytokine released in media with ELISA. Between every incubation step, several washes were performed. The amount of TNF-α (ng/mg protein) release was normalized to the protein content.

### Statistical analyses

Differences across the different treatments were identified using one-way ANOVA followed by Dunnett’s multiple comparisons test. The error bars represent the standard error of the mean (SEM).

## Results

### Astroglial cultures

When bought from the supplier, the astrocyte cultures contained a small percentage of microglial cells, less than 5%. Our experiments revealed the necessity of a specific number of microglia to obtain reactive inflammatory astrocytes. This number slightly increased after incubation with LPS for 24 h [29].

In the present study the induced Ca^2+^ release in the control cultures is unusually high compared to the induced Ca^2+^ release in LPS treated cells. This effect has been observed several times and can under certain circumstances be a problem. One explanation may be that the rats have some type of low-grade infection, virus, bacteria, and therefore express a high inflammatory level before the cultures are prepared. However, the main finding of this study is that inflammation is reduced in the LPS treated cells with our drug combination, which is clearly shown in the results.

### Restoration of inflammatory reactive astrocytes

The cultures were first incubated with LPS for 24 h to achieve inflammatory reactivity. For restoration of a normal physiological level, the cells remained in culture with LPS plus high glucose and different combinations of pharmaceuticals for an additional 24 h.

**Fig 1:**
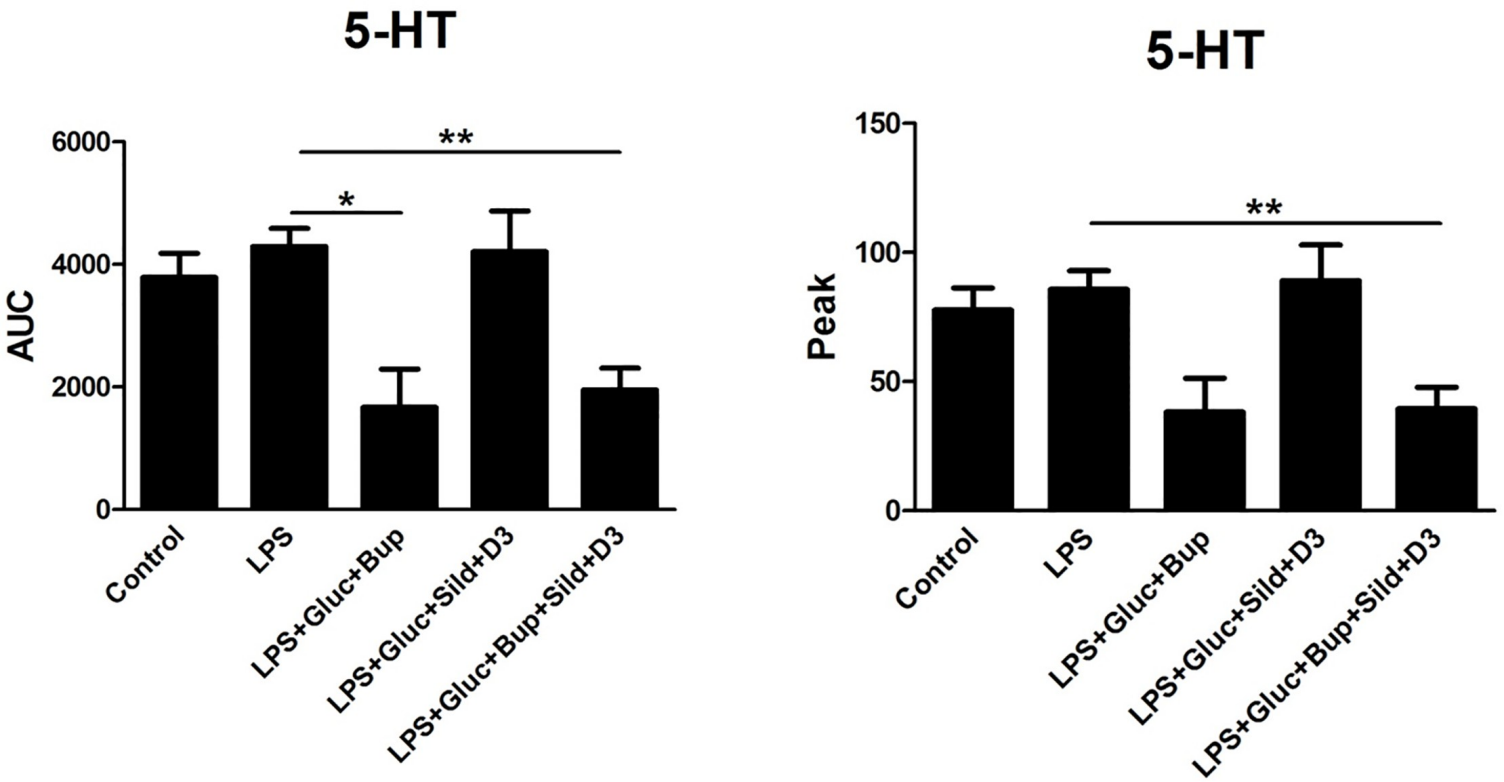
Astrocytes were incubated with the Ca^2+^-sensitive probe FLIPR Calcium 6. 5-HT (10^−5^ M) was used as a stimulator to detect changes in intracellular Ca^2+^ release. The cells were cultivated in 5.5 mM glucose for the entire cultivation period. Ca^2+^ responses were measured after the cells were stimulated with 5-HT (control), incubated with LPS (10 ng/ml) for 24 h, or incubated with LPS for 24 h followed by LPS, 25 mM glucose + bupivacaine (Bup) (10^−12^ M), 25 mM glucose + sildenafil (Sild) (1 μM) + vitamin D3 (D3) (100 nM), or a combination of all substances for an additional 24 h. The area under the curve (AUC) of the Ca^2+^ peak was calculated for each Ca^2+^ transient, and the amplitude (peak) was expressed as the maximum increase. The cells were obtained from 4 experiments with quadruple wells in each. The level of significance was calculated against LPS and was analyzed using a one-way ANOVA followed by Dunnett’s multiple comparisons test. * *P*<0.05, ** P< 0.01.

**Fig 2:**
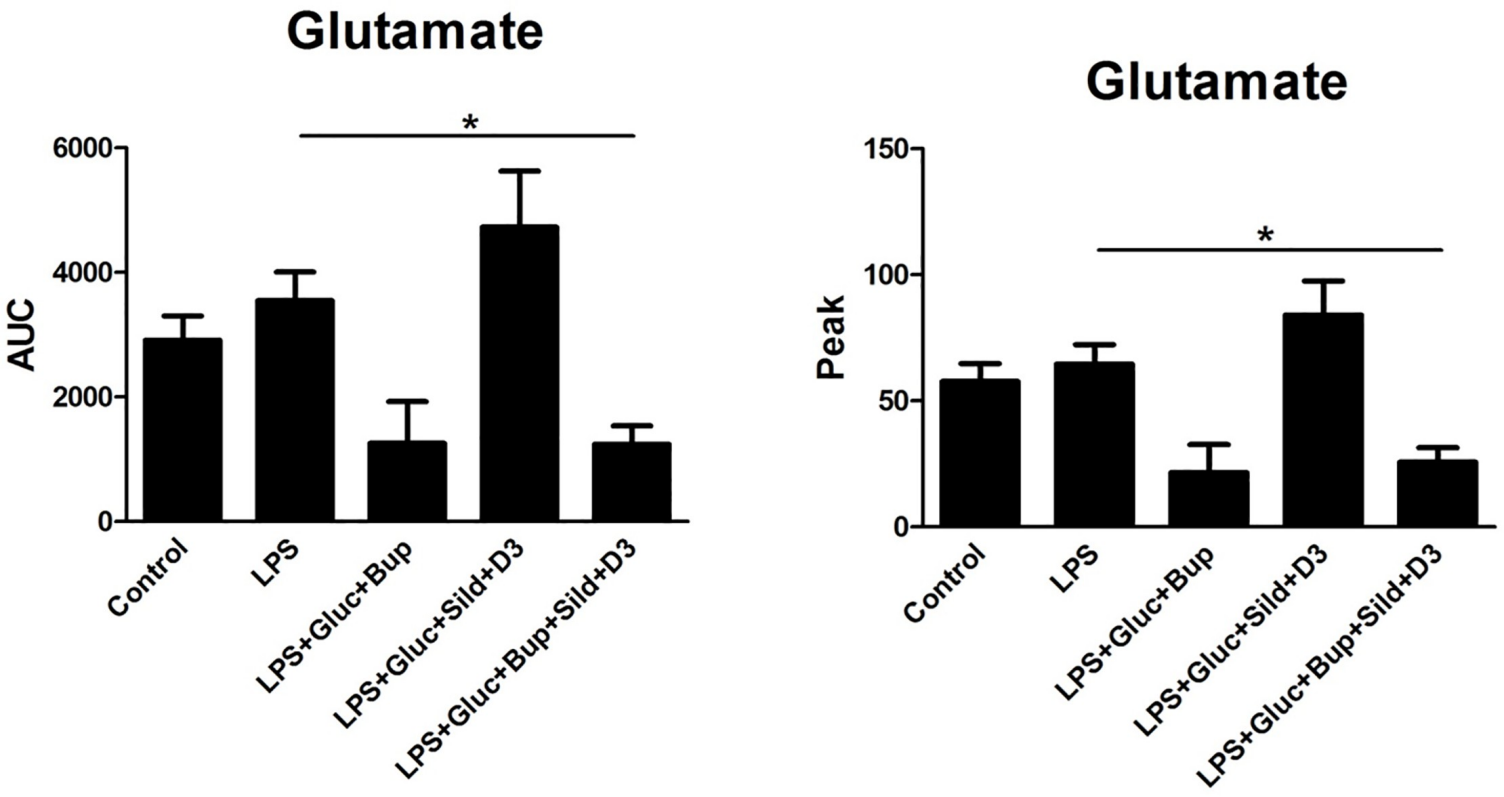
Astrocytes were incubated with the Ca^2+^-sensitive probe FLIPR Calcium 6. Glutamate (10^−3^ M) was used as a stimulator to detect changes in intracellular Ca^2+^ release. The cells were cultivated in 5.5 mM glucose for the entire cultivation period. Ca^2+^ responses were measured after the cells were stimulated with glutamate (control), incubated with LPS (10 ng/ml) for 24 h, or incubated with LPS for 24 h followed by LPS, 25 mM glucose + bupivacaine (Bup) (10^−12^ M), 25 mM glucose + sildenafil (Sild) (1 μM) + vitamin D3 (D3) (100 nM), or a combination of all substances for an additional 24 h. The area under the curve (AUC) of the Ca^2+^ peak was calculated for each Ca^2+^ transient, and the amplitude (peak) was expressed as the maximum increase. The cells were obtained from 4 experiments with quadruple wells in each. The level of significance was calculated against LPS and was analyzed using a one-way ANOVA followed by Dunnett’s multiple comparisons test. * *P*<0.05.

**Fig 3:**
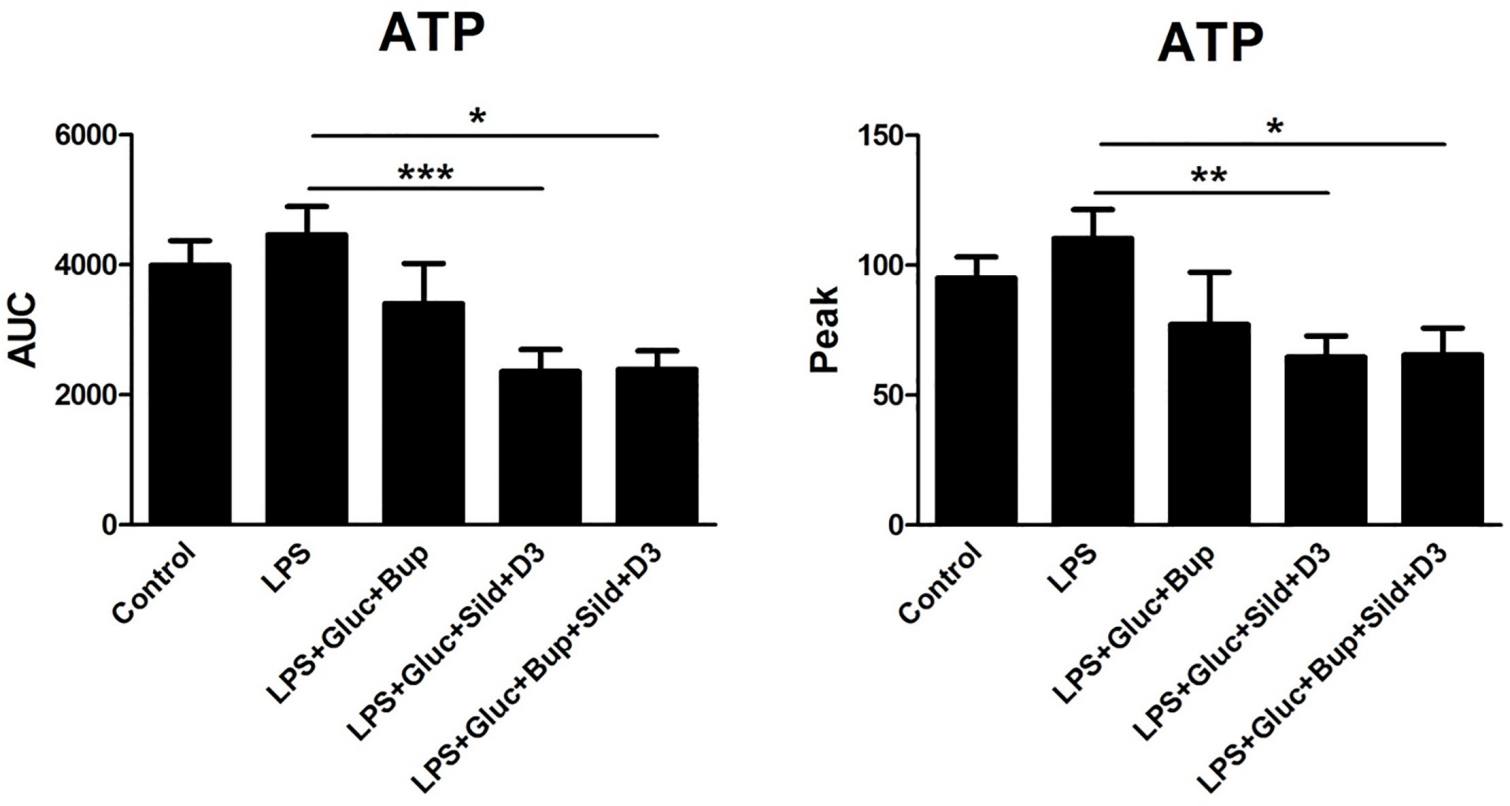
Astrocytes were incubated with the Ca^2+^-sensitive probe FLIPR Calcium 6. ATP (10^−4^ M) was used as a stimulator to detect changes in intracellular Ca^2+^ release. The cells were cultivated in 5.5 mM glucose for the entire cultivation period. Ca^2+^ responses were measured after the cells were stimulated with ATP (control), incubated with LPS (10 ng/ml) for 24 h, or incubated with LPS for 24 h followed by LPS, 25 mM glucose + bupivacaine (Bup) (10^−12^ M), 25 mM glucose + sildenafil (Sild) (1 μM) + vitamin D3 (D3) (100 nM), or a combination of all substances for an additional 24 h. The area under the curve (AUC) of the Ca^2+^ peak was calculated for each Ca^2+^ transient, and the amplitude (peak) was expressed as the maximum increase. The cells were obtained from 4 experiments with quadruple wells in each. The level of significance was calculated against LPS and analyzed using a one-way ANOVA followed by Dunnett’s multiple comparisons test. * *P*<0.05, ** P< 0.01, ***P<0.001.

An ultralow concentration of bupivacaine, previously evaluated with a concentration curve [17], attenuated 5-HT-evoked intracellular Ca^2+^ release in combination with high glucose (Fig 1). Neither glutamate- nor ATP-evoked intracellular Ca^2+^ release was affected by bupivacaine and high glucose (Figs 2 and 3).

The potent, selective PDE-5 inhibitor sildenafil induces cyclic GMP accumulation, which inhibits inflammation [27]. An extremely low concentration of sildenafil, 1 μM, is optimal [28]. Sildenafil in combination with vitamin D3 and high glucose attenuated the ATP-evoked intracellular Ca^2+^ release (Fig 3). Neither 5-HT- nor glutamate-evoked intracellular Ca^2+^ release was affected (Figs 1 and 2).

The combination of high glucose with vitamin D3 and ultralow concentrations of bupivacaine and sildenafil restored the 5-HT-, glutamate- and ATP-evoked intracellular Ca^2+^ release in inflammatory LPS-induced astrocytes to normal physiological levels (Figs 1–3).

**Fig 4:**
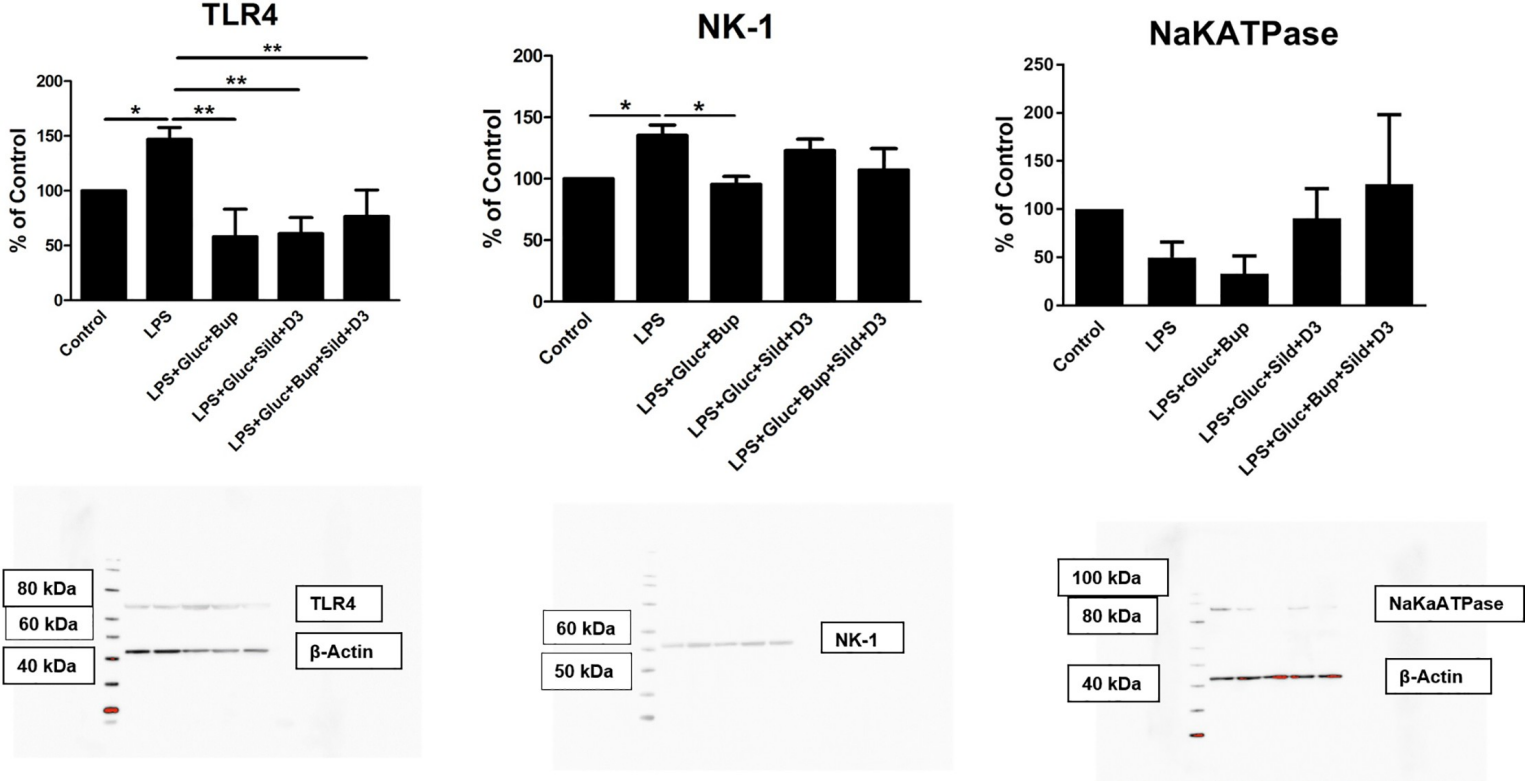
The expression levels of TLR4, NK-1 and Na^+^/K^+^-ATPase were studied using western blot analysis. Astrocytes were cultivated in 5.5 mM glucose for the entire cultivation period. The cells were incubated with LPS (10 ng/ml) for 24 h or incubated with LPS for 24 h followed by LPS, 25 mM glucose + bupivacaine (Bup) (10^−12^ M), 25 mM glucose + sildenafil (Sild) (1 μM) + vitamin D3 (D3) (100 nM), or a combination of all substances for an additional 24 h. The level of significance was calculated against LPS and analyzed using one-way ANOVA followed by Dunnett’s multiple comparisons test. *P< 0.05, ** P< 0.01. n = 6. Representative images of western blot membranes are presented.

TLR4 expression decreased with all drug combinations (Fig 4). NK-1 receptor expression was attenuated with bupivacaine and high glucose (Fig 4). The expression of Na^+^/K^+^-ATPase was not significantly increased (Fig 4).

**Fig 5:**
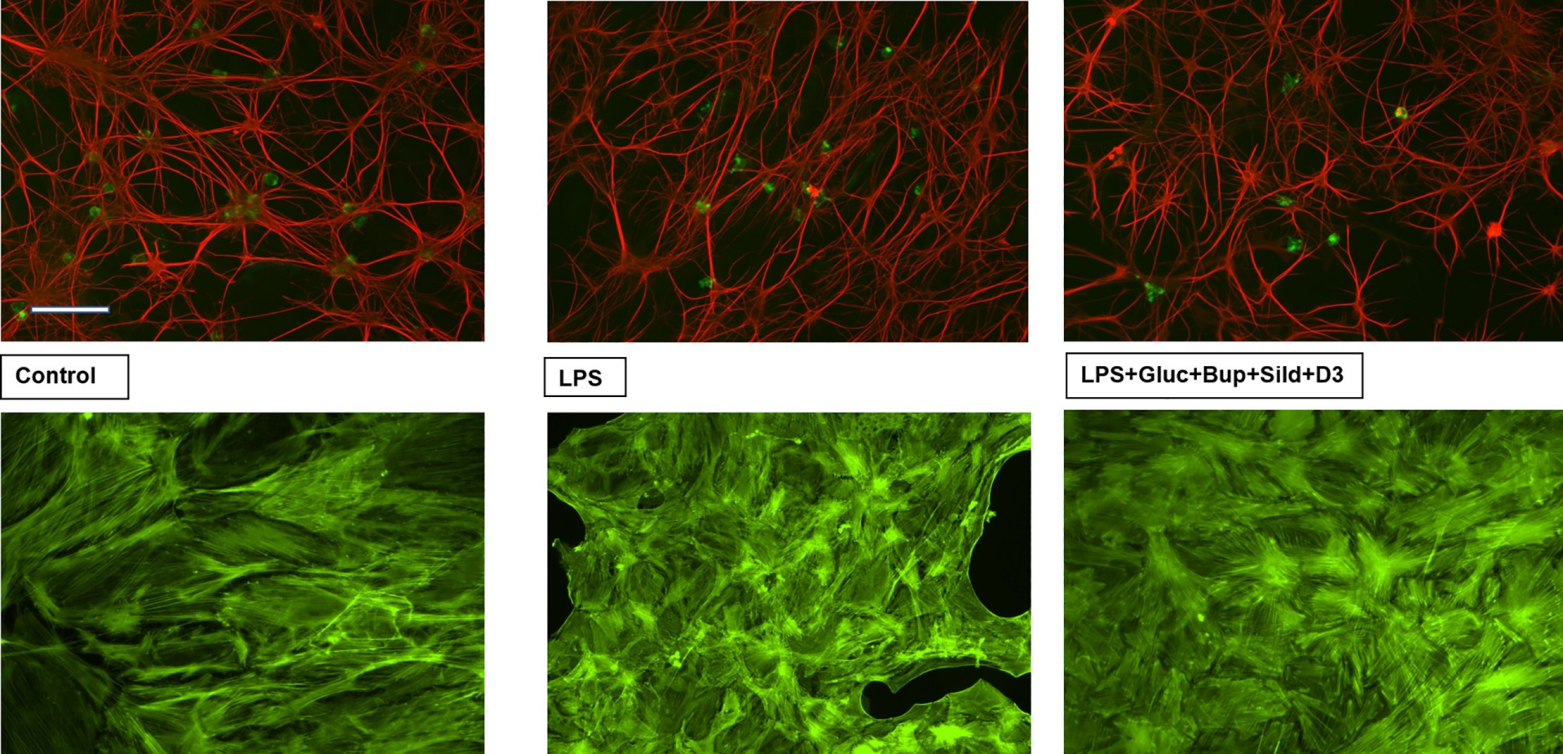
Cultures stained for the astrocytic marker GFAP (red) and the microglial marker OX42 (green), upper figures. Actin filament staining with Alexa488-conjugated phalloidin which shows mainly stress fibers in the control culture and after restoration while cells treated with LPS show a diffuse reorganization of the stress fibers, lower figures. Scale bar = 50 μm. Representative images are presented.

Control cultures showing astrocytes positive for GFAP and a few microglia positive for OX42. LPS treated cultures had some more microglia. Cultures treated with LPS and the combination of high glucose, bupivacaine, sildenafil and vitamin D3 show the same appearance as the control (Fig 5). The actin filaments were mainly organized in stress fibers in the control cultures, but changed when the cells were treated with LPS for 24 h. Restoration with the combination restored the morphology (Fig 5).

**Fig 6:**
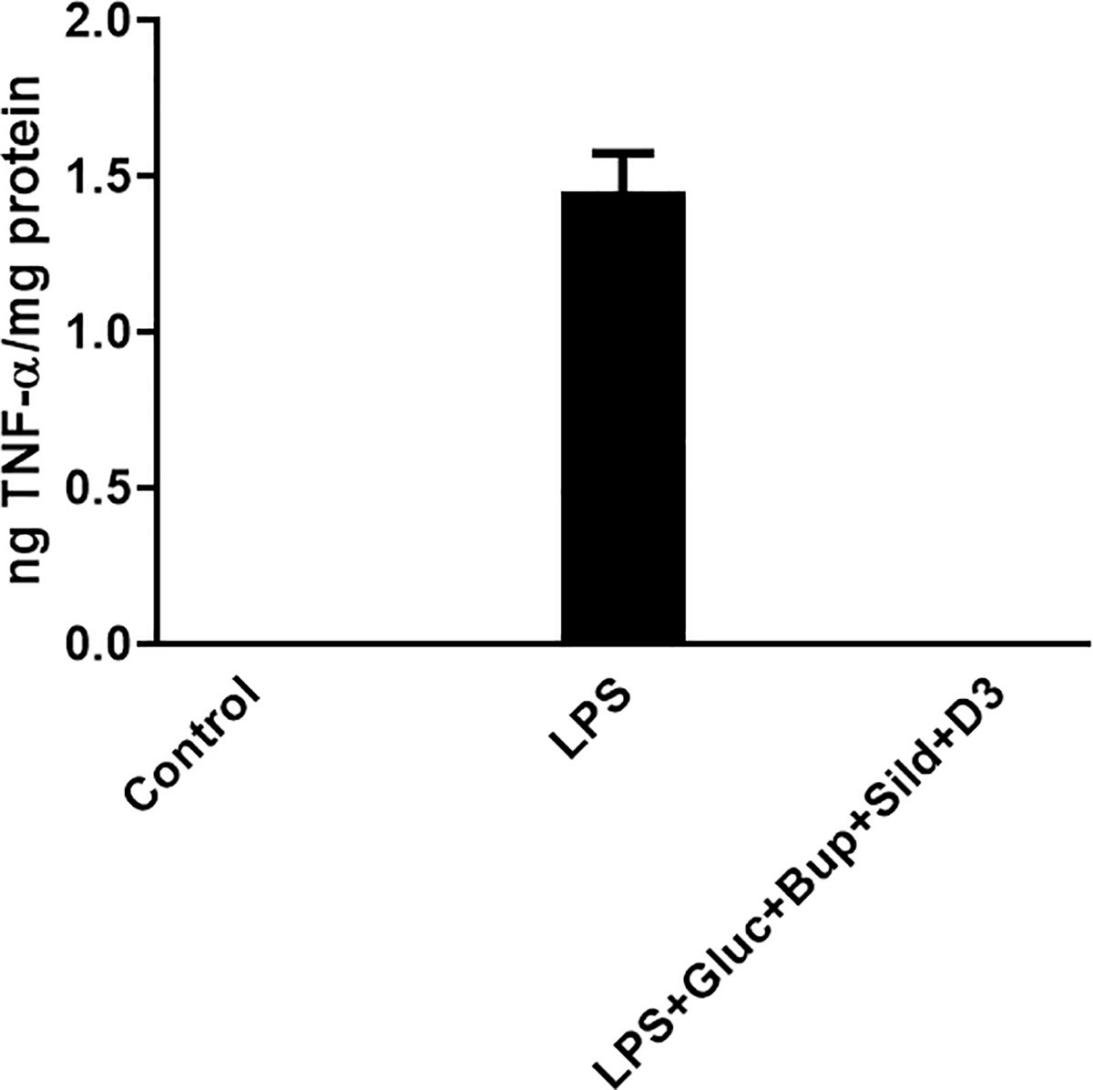
TNF-α (ng/mg protein) release observed after treatment with LPS for 24 h. No release was obtained either in controls or in cultures after restoration. n = 4.

Release of the pro-inflammatory cytokine TNF-α was observed neither in controls nor after restoration with the drug combination. TNF-α release was only obtained after LPS incubation for 24 h (Fig 6).

## Discussion

Astrocytes are gap junction-coupled cells in the nervous system, and these cellular networks have long been proposed to be targets in inflammatory processes [1,5,12,35–37]. The identification of anti-inflammatory substances that restore inflammatory reactive cells to a more physiological state has been difficult to discover. Many pharmaceutical substances have been proposed and tested over the years, many of which have been very promising at the experimental stage. However, only some of these substances have been tested in the clinic. We have continued to develop and further investigate a number of these substances. Some promising anti-inflammatory substances are the combination of a μ-opioid agonist, endomorphin-1/morphine/(-)-linalool, a μ-opioid antagonist, (-)-naloxone at an ultralow concentration, and the antiepileptic drug levetiracetam [8,38], which has been clinically tested with promising results [39]. Based on our findings, the Ca^2+^ signaling pathways can be stimulated by different neurotransmitters, such as ATP, 5-HT and glutamate. However, this triple combination showed effects on glutamate-evoked Ca^2+^ signaling only. Sildenafil has anti-inflammatory effects at very low concentrations [27,29,30,40], and by testing the substance at different concentrations, we found that sildenafil attenuated ATP-evoked intracellular Ca^2+^ release at low concentrations [28]. The combination of sildenafil and the above drugs particularly affects glutamate- and ATP-evoked Ca^2+^ signaling [29]. Thus, we hypothesize that this combination can have positive effects on neuroinflammatory diseases, but these effects must be tested in vivo before any further conclusions can be made.

Previous studies have shown that 5-HT-evoked intracellular Ca^2+^ release increases when astrocytes have been treated with LPS [41]. The purpose of the present study was to reduce 5-HT-evoked Ca^2+^ signaling in astrocytes that were treated with LPS to induce inflammatory reactivity.

Earlier studies [17] revealed that bupivacaine has at least two different mechanisms of action: anti-inflammatory properties at extremely low concentrations and anesthetic properties at high concentrations. In high concentrations, bupivacaine is used as a local anesthetic agent that blocks voltage-gated Na^+^ channels [42]. However, bupivacaine has other effects at much lower concentrations where blocking of Na^+^ channels does not occur [17]. At very low concentrations, bupivacaine evokes intracellular Ca^2+^ transients [17], and the enhanced 5-HT-evoked intracellular Ca^2+^ release in inflammatory reactive astrocytes is decreased by bupivacaine.

Restoration processes seem to have even better effects when cells are incubated in high-glucose conditions. High glucose has some anti-inflammatory effects [29,30]. In the present study, bupivacaine at extremely low concentrations in the presence of high glucose attenuated 5-HT-evoked intracellular Ca^2+^ release in LPS-induced inflammatory astrocytes. The expression levels of the inflammatory receptor TLR4 and the substance P receptor NK-1 also decreased.

Some single clinical tests have been performed with the combination of extremely low bupivacaine concentrations and high-glucose concentrations. This combination resulted in partially positive effects (unpublished).

However, we believe that ATP-evoked Ca^2+^ signaling has significant effects on inflammatory cells because ATP-evoked Ca^2+^ signaling increases substantially [28]. Since sildenafil has an effect on ATP-evoked Ca^2+^ signaling at extremely low concentrations, we wanted to test its effects together with bupivacaine on gap junction-coupled astrocytes. ATP is involved in all cellular signaling systems, and increased ATP production results in increased release from inflammation-induced astrocytes [10,38,43,44]. ATP stimulates purinergic receptors on astrocytes, thereby inducing increased Ca^2+^ signaling, which has also been observed in inflammatory diseases [11].

During inflammation, inflammatory receptors such as TLR4 and NK-1 increase in expression, and BBB disruption occurs [4]. We searched for a substance with properties to suppress the expression of inflammatory receptors and any substance that could reduce the damage to the BBB. Interestingly, vitamin D3 has these properties [22,23,45]. In addition, astrocytes express the vitamin D3 receptor VDR [46].

We found that restoration with sildenafil, vitamin D3 and high glucose reduced ATP-evoked Ca^2+^ signaling in LPS-induced astrocytes. TLR4 expression was also decreased. However, this combination had no effect on 5-HT-evoked Ca^2+^ signaling. Therefore, we combined the following substances: bupivacaine, sildenafil (both at extremely low concentrations), vitamin D3 and high glucose. Surprisingly, 5-HT-evoked Ca^2+^ signaling, ATP-evoked Ca^2+^ signaling, and glutamate-evoked Ca^2+^ signaling were attenuated.

The purpose of our chosen interventions was to affect 5-HT- and ATP-evoked intracellular Ca^2+^ release, which was achieved with the combination of vitamin D3 and ultralow concentrations of both bupivacaine and sildenafil in high-glucose medium. Moreover, glutamate-evoked intracellular Ca^2+^ release was attenuated, and TLR4 expression decreased with all combinations.

## Conclusions

Based on these unexpected results, we conclude that the pharmaceutical combination of bupivacaine and sildenafil (both in extremely low concentrations), together with vitamin D3 and high glucose, is needed to aid inflamed cells in regaining homeostasis by restoring 5-HT-, ATP-, and glutamate-evoked intracellular Ca^2+^ release, thereby downregulating the inflammatory status. Our results show that several vital reversible cellular functions in inflammatory reactive gap junction-coupled astrocytes can be fully restored with a combination of pharmaceuticals. However, the concentration of each individual substance is important to obtain the appropriate cellular effects. Future studies will continue to investigate the in vivo effects of this pharmaceutical combination in preclinical and clinical trials.

## Abbreviations

PDE-5: phosphodiesterase-5
vitamin D3: 1α,25-Dihydroxyvitamin D3
TLR4: Toll-like receptor 4
NK-1: Substance P receptor
CNS: central nervous system
BBB: blood-brain barrier
TNF-α: tumor necrosis factor-α
IL-1β: interleukin-1β
IL-6: interleukin-6
LPS: lipopolysaccharide
VDR: vitamin D receptor
iNOS: nitric oxide synthase
NO: nitric oxide
PKG: protein kinase G
PDEs: phosphodiesterases
AMPK: AMP-activated protein kinase
PBS: phosphate-buffered saline
RIPA: radio-immunoprecipitation assay
